# Supplementary material for: A Commonly Used Photosynthetic Inhibitor Fails to Block Electron Flow to Photosystem I in Intact Systems
Source: Front Plant Sci. 2020 Apr 15;11:382. doi: 10.3389/fpls.2020.00382 (PMC7174583; doi:10.3389/fpls.2020.00382)

Figure. S1. A response of various concentrations of DNP-INT on (A) PC redox kinetics, (B) P700 redox kinetics in isolated thylakoids. All measurement condition and light intensity were similar as explained in Figure. 2. For measurements in Dual-Klass NIR, the isolated thylakoids (control or inhibitor treated) equivalent to 200 µl (1 mg Chl ml-1) was smeared gently on a filter paper (Whatman filter paper 55 mm, thickness 390 µM) and covered with another filter paper of the same thickness. The filter papers were put under a clean transparent petri dish and moistened with 1000 µl of measurement buffer alone or containing desired concentrations of inhibitors. The P700 redox kinetics was measured from these isolated thylakoids, sandwiched between thin filter papers on Dual-Klass-NIR.

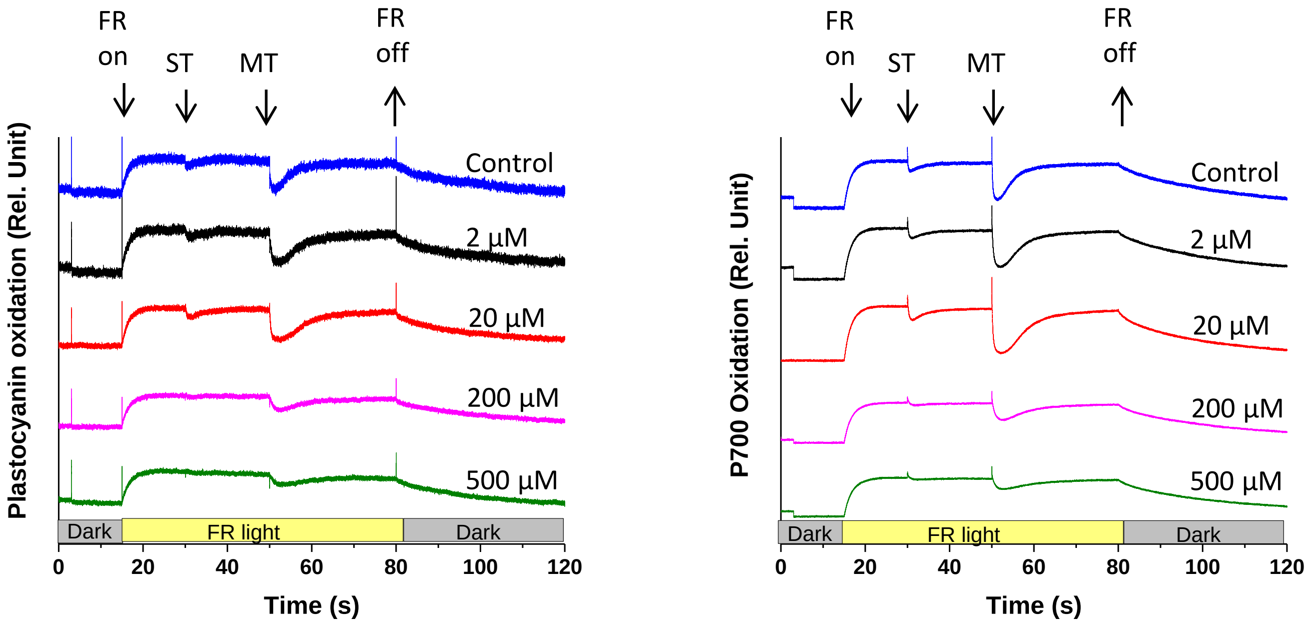

Supplement: Supplementary file 1 [file Data_Sheet_1.PDF]
