# Supplementary material for: A Commonly Used Photosynthetic Inhibitor Fails to Block Electron Flow to Photosystem I in Intact Systems
Source: Front Plant Sci. 2020 Apr 15;11:382. doi: 10.3389/fpls.2020.00382 (PMC7174583; doi:10.3389/fpls.2020.00382)

Figure S3. Photosystem I redox kinetics in intact leaf and isolated thylakoid samples with DNP-INT synthesized by Prof. Achim Trebst. P700 redox kinetics were measured with Dual Pam in intact leaves (Dotted lines), and isolated thylakoids (solid line). The measurement conditions are as described in legends of figure 2.

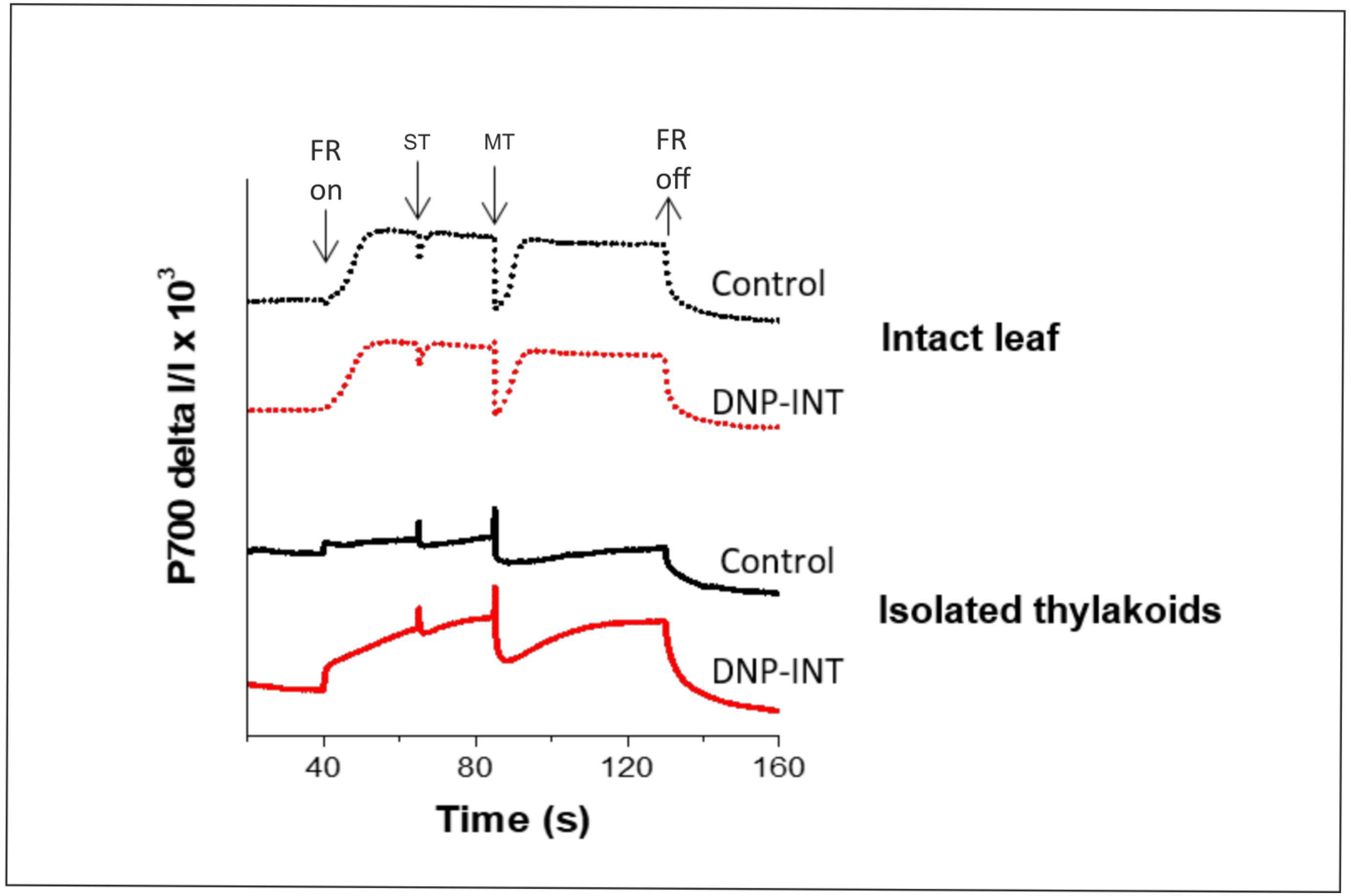

Supplement: Supplementary file 3 [file Data_Sheet_3.PDF]
