# Supplementary figures and images for: A Commonly Used Photosynthetic Inhibitor Fails to Block Electron Flow to Photosystem I in Intact Systems
Source: Front Plant Sci. 2020 Apr 15;11:382. doi: 10.3389/fpls.2020.00382 (PMC7174583; doi:10.3389/fpls.2020.00382)

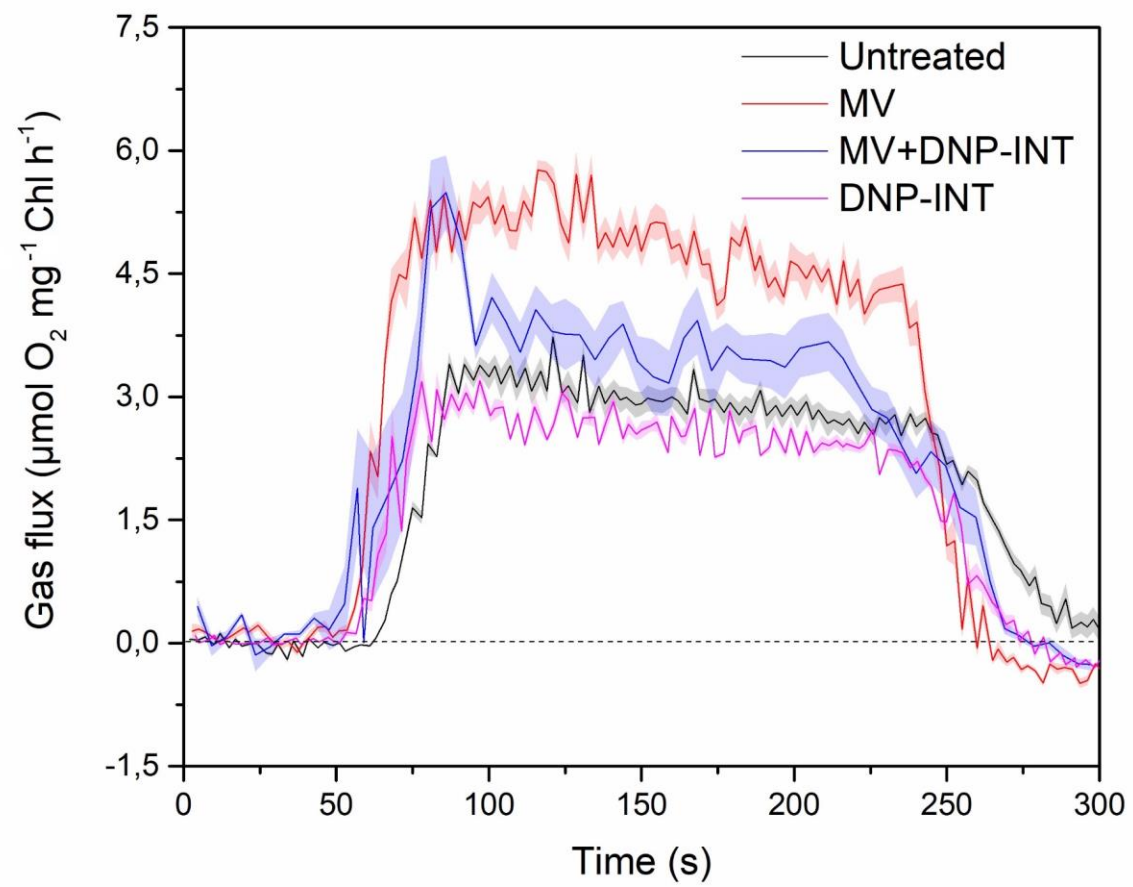

Figure S4. A comparison of gross oxygen evolution rates from fig. 4.

Supplement: Supplementary file 4 [file Data_Sheet_4.PDF]
