# Supplementary material for: A Commonly Used Photosynthetic Inhibitor Fails to Block Electron Flow to Photosystem I in Intact Systems
Source: Front Plant Sci. 2020 Apr 15;11:382. doi: 10.3389/fpls.2020.00382 (PMC7174583; doi:10.3389/fpls.2020.00382)
Supplement: Supplementary file 2 [file Data_Sheet_2.PDF]

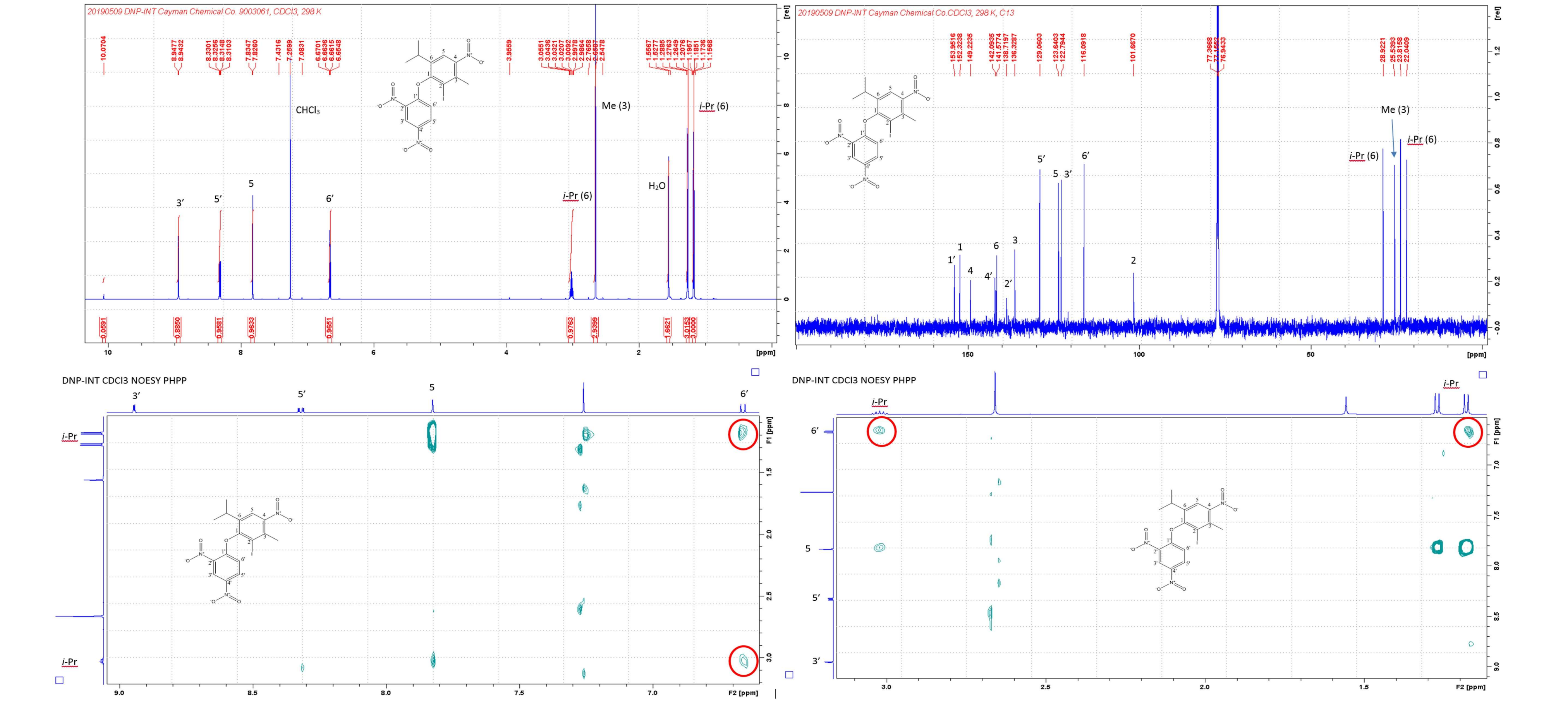

Figure S2 The NMR spectra were recorded at 298 K temperature. Experiment temperature is uncorrected. The chemical shifts were referenced internally to solvent signal, appropriate 2D NMR methods, *i.e.* HMBC and HSQC, were used for peak assignment * *H. E.Gottlieb, V. Kotlyar, and A. Nudelman, *J. Org. Chem.,* 1997, **62**, 7512–7515.

NOE interaction between isopropyl group and the proton 6′ of the second aromatic ring proves that the rings are connected.

^{1}H NMR (600 MHz, $CDCl_3$): δ = 1.18 (d, ^{3}J = 6.9 Hz, 3 H), 1.27 (d, ^{3}J = 6.8 Hz, 3 H), 2.65 (s, 3 H), 3.02 (m, ^{3}J = 6.9 Hz, 1 H), 6.66 (d, ^{3}J = 9.2 Hz, 1 H), 7.83 (s, 1 H), 8.32 (dd, ^{3}J = 9.2 Hz, ^{4}J = 2.7 Hz, 1 H), 8.95 (d, ^{4}J = 2.7 Hz, 1 H) ppm;

^{13}C NMR (151 MHz, $CDCl_3$): δ = 22.04, 23.82, 25.54, 28.92, 101.67, 116.09, 122.79, 123.64, 129.06, 136.33, 138.72, 141.58, 142.09, 149.22, 152.32, 153.95 ppm.
